# Combined transepithelial phototherapeutic keratectomy and corneal collagen cross-linking for corneal ectasia after small-incision lenticule extraction—preoperative and 3-year postoperative results: a case report

**DOI:** 10.1186/s12886-018-0849-4

**Published:** 2018-07-17

**Authors:** Qingman Ge, Chuanbo Cui, Jing Wang, Guoying Mu

**Affiliations:** 1 Department of Ophthalmology, Shandong Provincial Hospital affiliated to Shandong University, HuaiYin District, Jing 5 Wei 6 Road no. 324, Jinan, China; 2 Lunan Eye Hospital, Lanshan District, Yucai Road no. 109, Linyi, China; 3 Department of Ophthalmology, Shandong Medical College, Lanshan District, Jucai Road no. 6, Linyi, China

**Keywords:** Corneal collagen, Corneal ectasia, Cross-linking, Phototherapeutic keratectomy, Small-incision lenticule extraction

## Abstract

**Background:**

Corneal ectasia after small-incision lenticule extraction (SMILE) is uncommon. To our knowledge, this is the first report of 3-year results of combined phototherapeutic keratectomy (PTK) and corneal collagen cross-linking (CXL) for corneal ectasia after SMILE.

**Case presentation:**

Herein, we describe a case of prominent corneal ectasia after SMILE treated with PTK combined with CXL 3 years ago. After surgery, maximum corneal keratometry, mean corneal keratometry, spherical equivalent and uncorrected distance visual acuity were significantly improved at follow-up intervals.

**Conclusions:**

Transepithelial PTK combined with CXL for corneal ectasia after SMILE may be an effective and safe treatment in the long term.

## Background

Corneal ectasia after small-incision lenticule extraction (SMILE) is uncommon. While in theory SMILE preserves a stronger cornea postoperatively, there are some previous reports of ectasia developing after SMILE [1–5]. We have never seen any treatment about corneal ectasia after SMILE. Herein, we describe a case of prominent corneal ectasia after SMILE that was treated with phototherapeutic keratectomy (PTK) combined with corneal collagen cross-linking (CXL).

## Case presentation

**Fig. 1:**
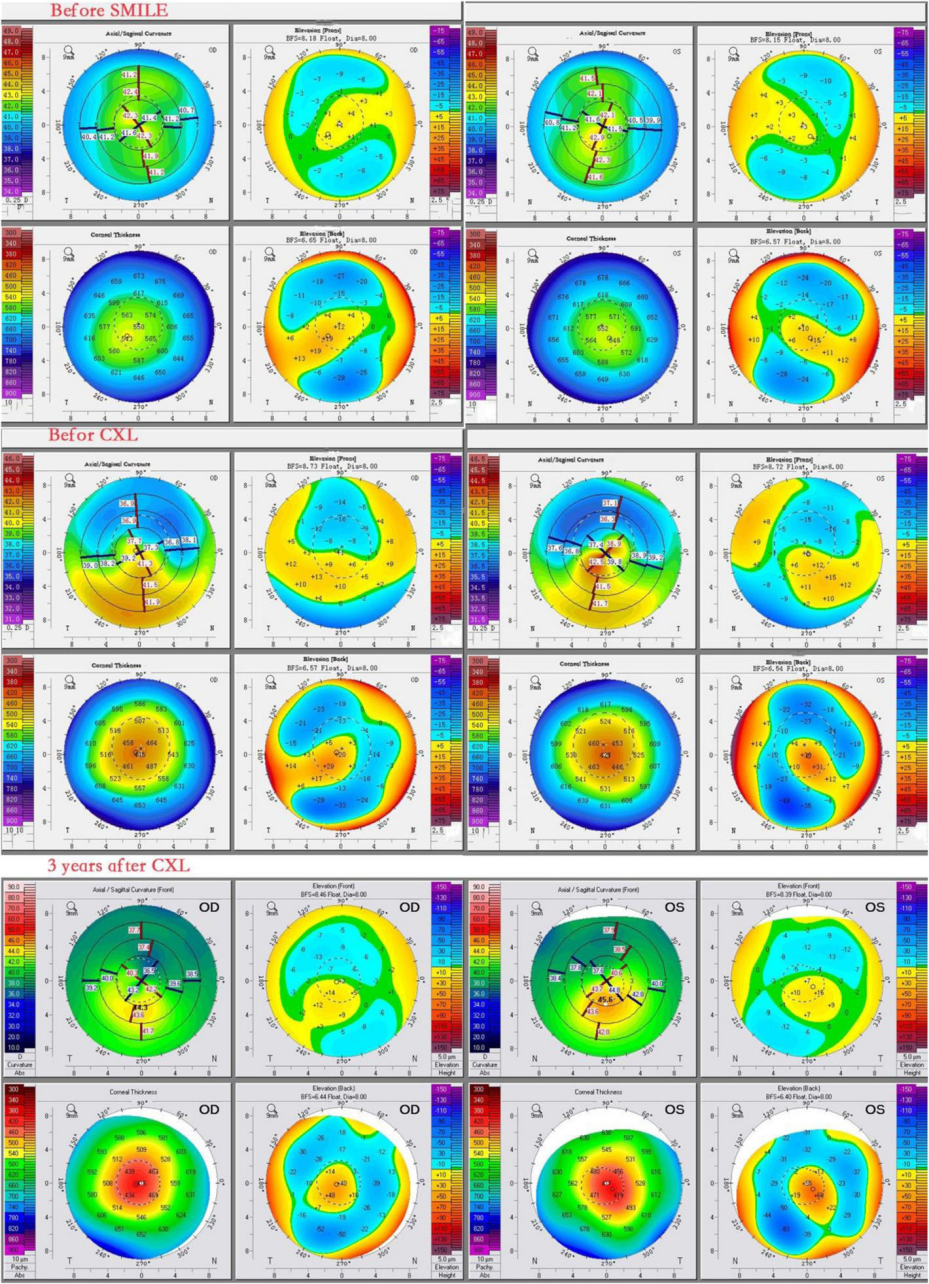
Corneal topography before SMILE, before CXL and at 3 years after CXL

In June 2013, a 19-year-old male patient underwent SMILE for myopia in both eyes. He had a history of eye rubbing and allergic conjunctivitis, and before SMILE he had no history of pellucid marginal corneal degeneration and no family history of keratoconus or high myopia. Preoperative characteristics and parameters are summarized in Table 1. Preoperative topographies are presented in Fig. 1. The manifest refraction values were − 6.75 DS with 1.00 DC × 45 in the right eye and − 6.75 DS with 0.75 DC × 140 in the left eye, and corrected distance visual acuity (CDVA) was 20/20 in both eyes.

**Table 1:** Preoperative and postoperative characteristics, parameters and topographies

Follow-up	eye	Kmean (D)	Kmax (D)	TCT (μm)	Spherical equivalent (D)	UDVA (Snellen)
pre-SMILE	OD	42.00	43.00	546.00	−7.25	20/100
	OS	42.30	44.30	542.00	−7.00	20/80
post-SMILE month 1	OD	37.60	41.90	433.00	−0.25	20/20
	OS	37.80	41.30	429.00	−0.50	20/20
post-SMILE month 7.5	OD	38.90	42.20	445.00	−1.25	20/20
	OS	39.70	44.00	426.00	−1.75	20/20
post-SMILE month 14	OD	41.80	46.90	432.00	−4.25	20/32
	OS	43.20	49.60	412.00	−5.25	20/40
post-CXL month 6	OD	40.00	46.40	389.00	−3.50	20/25
	OS	41.20	48.30	358.00	−4.25	20/32
post-CXL month 12	OD	39.80	45.50	407.00	−3.00	20/22
	OS	41.20	47.60	393.00	−4.00	20/30
post-CXL month 24	OD	40.20	43.90	415.00	−3.00	20/22
	OS	41.30	45.70	402.00	−3.75	20/27
post-CXL month 36	OD	40.30	44.30	412.00	−2.75	20/22
	OS	41.60	45.60	408.00	−3.50	20/25

*Kmean* mean corneal keratometry, *Kmax* maximum corneal keratometry, *TCT* thinnest corneal thickness, *UDVA* uncorrected distance visual acuity, *SMILE* small-incision lenticule extraction, *CXL* corneal collagen cross-linking

At 1 month after SMILE, uncorrected distance visual acuity (UDVA) was 20/20 in both eyes. The patient had no complaints of a decline in vision. Corneal topography revealed corneal ectasia. Posterior elevation was + 21 μm in both eyes, and respective thinnest corneal thicknesses (TCTs) were 433 mm and 429 mm in the right and left eyes. The patient’s characteristics, parameters and topographies are presented in Table 1 and Fig. 1. Pachymetry examination showed a decentred elevation coincident with the thinnest point on the posterior surface.

At 7.5 months, topography indicated posterior elevation of + 29 μm in the right eye and + 31 μm in the left (Table 1, Fig. 1). Respective TCTs were 445 mm and 426 mm in the right and left eyes. At 14 months after SMILE, UDVA had reduced to 20/32 in the right eye and 20/40 in the left (Table 1, Fig. 1). The patient still exhibited eye rubbing and intermittent episodes of allergic conjunctivitis after SMILE. Simultaneous PTK and CXL (PTK + CXL) was performed in both eyes. Before CXL, the central 9.0-mm diameter epithelium with a 50 μm depth was removed via PTK using the excimer laser system (EX500, Wavelight Technologies, Germany). The general CXL technique used was based on the original CXL procedure described by Wollensak et al. [6]. riboflavin solution (0.1%)was administered topically every 2 min for a total of 30 min. The cornea was then irradiated with ultraviolet A light (370 nm, 3.0 mW/cm^2^) (UV-X illumination system version 1000, UVXTm, IROCAG, Zurich, Switzerland) for 30 min at a distance of 5 cm, with continuous application of the riboflavin solution every 2 min. At the end of the procedure, a soft bandage contact lens was placed until corneal reepithelialisation had occurred. Antibiotic eye drops were administrated 4 times daily for 1 week, and fluorometholone eye drops 0.1% were administrated 4 times daily for 4 weeks. No intraoperative or postoperative complications occurred. The follow-up keratometry values and TCT, spherical equivalent and UDVA measurements at 6, 12, 24 and 36 months after PTK + CXL are summarized in Table 1.

At 36 months, in the right eye maximum corneal keratometry (Kmax) and mean corneal keratometry (Kmean) were decreased by 2.6 D and 1.5 D, respectively, while in the left eye the corresponding reductions were 4.0 D and 1.6 D. TCT had decreased from 432 μm to 412 μm in right eye, and had not changed in the left eye. Spherical equivalent improved significantly over the course of the study, from − 4.25 D to − 2.75 D in the right eye and from − 5.25 D to − 3.5 D in the left eye at 36 months. UDVA increased over time, and did not require glasses or enhancement.

## Discussion

Corneal ectasia after SMILE is an uncommon but a serious complication associated with a decline in biomechanical stability, and visual impairment. There are some reports of ectasia developing after SMILE [1–5]. To our knowledge however, this is the first report to include 3-year results of combined PTK + CXL for corneal ectasia after SMILE.

The cases of ectasia after SMILE that have been reported have exhibited abnormal preoperative topographic patterns. When corneal topography revealed corneal ectasia after SMILE, we reviewed the corneal topography acquired before SMILE and observed a decentred elevation coincident with the thinnest point on the posterior surface, and a slight asymmetry on the anterior surface. We then made a retrospective diagnosis of forme fruste keratoconus. Therefore, we suggest that forme fruste keratoconus should be regarded as a possible risk factor for ectasia after SMILE, and the same preoperative topographic and tomographic screening criteria that we have developed could be used for laser-assisted in situ keratomileusis (LASIK) and PRK [7]. The application of strict screening criteria may prevent this phenomenon in patients undergoing SMILE.

Postoperative corneal ectasia is most likely associated with a reduction in the biomechanical integrity below the threshold required to maintain corneal shape [8]. Xia et al. [9] reported that the postoperative corneal hysteresis and corneal resistance factor values were significantly lower than the preoperative values in a group of SMILE patients. More specifically, in the SMILE group in another study the average tensile strength reduction for − 3.00 D correction was − 34.46% compared to the control, and for − 8.00 D correction it was − 49.34% compared to the control [10]. The patient in the current report had a history of eye rubbing and allergic conjunctivitis before SMILE, and these conditions remained after it. Liu et al. [11] performed an experiment involving an eye-rubbing test that included 2 episodes, each lasting 20 s, with a 2-min break between episodes. They found that corneal hysteresis and corneal resistance factor were both significantly reduced after the first episode of eye rubbing, and that after the second episode of eye rubbing they were significantly reduced even further. Thus, we believe that biomechanical weakening is the most important risk factor for corneal ectasia. We also speculate that eye rubbing and allergic conjunctivitis may have played an active role in corneal ectasia after SMILE in the current case.

CXL—a minimally invasive treatment—was introduced in 2003 as a new treatment modality to halt the progression of ectasia by inducing corneal cross-linking to increase the corneal elastic modulus and hence increase biomechanical stress resistance [6]. The procedure should be performed after the progression process is evident, as most surgeons suggest. Many studies have reported favourable results after using CXL to treat keratoconus and post-LASIK ectasia. With regard to the treatment of ectasia after LASIK, Hafezi et al. [12] reported reductions in Kmax at 12 months in all cases, and improved CDVA in 40% of cases. PTK epithelial removal at a constant depth removes some stromal tissue at the top of the cone in addition to overlying epithelium, thereby having a smoothing effect [13]. Transepithelial PTK + CXL has been gradually adopted, and has demonstrated some benefits with regard to halting progression while improving visual function [14]. Kapasi et al. [15] reported that Kmean had decreased by 2.01 D at 12 months follow-up using PTK + CXL for the management of keratoconus. In the current case, Kmax and Kmean were significantly reduced. The lowest Kmean value was observed at 6 months, and it remained stable between 12 and 36 months. Kmax declined gradually over the course of the 3-year follow-up. These results were consistent with Caporossi et al. [16, 17]. They reported that CXL improved functional performance 3 years after the operation in 80% of patients, and they also demonstrated a reduction of 0.57 D in Kmax over a 4-year period after CXL. Topographic results may continue to exhibit improvements at future follow-up time-points.

In the present case, spherical equivalent and UDVA exhibited significant improvements in both eyes at each time-point as compared to the corresponding values before CXL. These results are concordant with those of Gaster et al. [18], who reported that spherical equivalent improved from − 3.80 ± 3.90 to − 2.50 ± 3.10 D, and UDVA exhibited similar improvement from 20/142 Snellen to 20/83 Snellen at 24 months after transepithelial PTK + CXL for progressive keratoconus.

In the current patient, TCT exhibited a significant reduction at 6 months after CXL, then improved progressively over the next 30 months. This is similar to results in studies reported by Hashemi et al. [19] and Caporossi et al. [20], in which the mean CCT in the early stage of CXL was significantly lower than baseline, then subsequently increased significantly. Optical pachymetric data in the early postoperative stage was negatively affected by epithelial thinning, corneal suboedema, and keratocyte loss in the anterior–mid stroma [21, 22]. Mazzotta et al. [23] reported that epithelium was very thin (10–20 μm) 1 month after CXL in the apex region of keratoconus, and normal epithelium thickness resembling preoperative data was detected between 3 and 6 months after CXL.

## Conclusions

Surgeons should regard forme fruste keratoconus as a possible risk factor for ectasia after SMILE, and pay attention to eye rubbing and allergic conjunctivitis before refractive Surgery. This is the first case report describing a patient with corneal ectasia after SMILE treated by transepithelial PTK + CXL, which could be an effective and safe treatment in the long term as demonstrated herein over a 3-year follow-up period.

## Abbreviations

CDVA: Corrected distance visual acuity
CXL: Corneal collagen cross-linking
Kmax: Maximum corneal keratometry
Kmean: Mean corneal keratometry
LASIK: Laser-assisted in situ keratomileusis
PTK: Phototherapeutic keratectomy
SMILE: Small-incision lenticule extraction
TCT: Thinnest corneal thickness
UDVA: Uncorrected distance visual acuity
